# Teachers Helping EFL Students Improve Their Writing Through Written Feedback: The Case of Native and Non-native English-Speaking Teachers' Beliefs

**DOI:** 10.3389/fpsyg.2022.804313

**Published:** 2022-03-09

**Authors:** Xiaolong Cheng, Lawrence Jun Zhang

**Affiliations:** ^1^School of Foreign Languages, Hubei University of Technology, Wuhan, China; ^2^Faculty of Education and Social Work, University of Auckland, Auckland, New Zealand

**Keywords:** native English-speaking teachers, non-native English-speaking teachers, teachers' beliefs, written feedback, EFL writing

## Abstract

Although the efficacy of teacher written feedback has been widely investigated, relatively few studies have been conducted from feedback practitioners' perspectives to investigate teachers' beliefs regarding it, particularly compare beliefs held by teachers with different sociocultural and linguistic backgrounds. Consequently, much remains to be known about teachers' conceptions about written feedback, who has different first languages (L1). To bridge such a gap, we conducted this qualitative study to examine the similarities and differences between native English-speaking (NES) and non-native English-speaking (NNES) teachers' beliefs in Chinese University EFL settings. We analyzed the in-depth interviews with eight teachers through thematic analysis. The findings showed that NES and NNES teachers espoused a range of beliefs in relation to the five themes of written feedback: Purpose, scope, focus, strategy, and orientation. While they shared similar beliefs with regard to feedback focus, their beliefs differed in terms of feedback scope. Important implications are discussed for educational practices.

## Introduction

Writing in a second/foreign language poses a great challenge to both teachers and students (Zhang and Qin, 2018; Hyland and Hyland, 2019; Wei et al., 2020; Sun et al., 2021; Zhan et al., 2021; Zhang T. T. and Zhang L. J., 2021). This is because writing is commonly understood as the most demanding language skill among the language skills such as listening, speaking, reading and writing (Huang and Zhang, 2020; Zhang L. J, 2021). In the learning-to-write process, students expect teachers to offer them feedback and teachers do take feedback provision as one of the significant pedagogical procedures in teaching writing (Li et al., 2020; Teng and Zhang, 2020, 2021; Zhang and Zhang, 2020; Cheng and Zhang, 2021a,b). Feedback can be offered to students at the global or local levels depending on the learning task expected to be completed (Chen and Zhang, 2017; Rahimi and Zhang, 2021; Zhang et al., 2021). Embedded in the pedagogical contexts, feedback functions to facilitate students' learning process and improve their learning outcomes (Sadler, 1989; Hattie and Timperley, 2007; Zhang, 2018; Gan et al., 2021). As a crucial type of feedback and popular instructional activity, teacher written feedback is extensively employed in L2 writing instruction and it is believed to scaffold L2 writers' writing process as well as improve their L2 writing proficiency (Zhang, 2013; Hyland and Hyland, 2019). Recent years have witnessed the proliferation of studies about teacher written corrective feedback (WCF) (Zhang and Cheng, 2021; Zhang T. F., 2021). In the existing literature, there is a spirited controversy centering on WCF efficacy. It was triggered by Truscott (1996), who have cast doubt on the effectiveness of WCF. However, researchers, at present, appear to have reached a consensus that WCF benefits writing accuracy in L2 learners' revised/new writing (e.g., Bitchener and Knoch, 2009, 2010; Shintani and Aubrey, 2016; Li and Roshan, 2019; Karim and Nassaji, 2020).

Currently, copious research has concentrated on the relative effects of different types of WCF. Unfortunately, there are sparse descriptive studies examining teachers' conceptualizations of written feedback in their pedagogical settings (Lee, 2017; Yu et al., 2020; Zhang T. F., 2021). Furthermore, these inquiries, in general, do not occur in mainland China (Mao and Crosthwaite, 2019; Yu et al., 2020). Considering the profound impact of contexts on the formation and reformation of teachers' beliefs about teaching (Rahimi et al., 2016; Bao, 2019; Gao and Zhang, 2020; Sun and Zhang, 2021; Wu et al., 2021), more studies are warranted in the mainland Chinese EFL classrooms.

Moreover, with the advent of globalization, English serves as the lingua franca in the world (Rubdy et al., 2012). As such, numerous NES teachers are recruited to teach English in NNES countries annually (Rao and Li, 2017; Rao and Yu, 2021; Zhang J. H. and Zhang L. J., 2021). The growing number of NES teachers in these countries bring new insights into EFL teaching and motivate scholars to compare expatriate NES and local NNES teachers' teaching philosophies (Clark-Gareca and Gui, 2019). Whereas many NES teachers are responsible for EFL writing because of their genre awareness and good command of English writing conventions (Zhang, 2016; Rao and Li, 2017), little is known about how NES and NNES teachers conceptualize written feedback in their belief systems. Due to the fact that sociocultural and language backgrounds influence and mediate teachers' beliefs about written feedback (Lee, 2013, 2017; Hyland and Hyland, 2019), it is worthwhile for researchers to investigate such an issue.

To fill these research voids, we implemented an exploratory study to examine the similarities and differences between the two groups of teachers' written feedback beliefs in mainland Chinese EFL writing settings. This study is expected to extend our existing knowledge base of EFL teachers' beliefs about written feedback and provide some useful implications for such teachers' feedback practices, which has been a central issue in the scholarship on L2 writing (Zhang, 2013, 2016; Zheng and Yu, 2018).

## Literature Review

### Teacher Written Feedback

To date, researchers have addressed the typology of teacher written feedback. Derived from previous studies, this review identified several recurring themes in relation to teacher written feedback: Scope, focus, strategy, and orientation (e.g., Lee, 2008, 2009, 2013; Sheen, 2011; Bitchener and Ferris, 2012). Feedback focus is defined as what teachers focus on while responding to their students' written texts (Yu and Lee, 2014). Generally, teachers pay attention to different aspects of writing when giving feedback, including Language, content, and organization. Researchers further classified *language* into the local issues, while they considered *content* and *organization* as the global issues of writing (Butler and Britt, 2011). As a result, we can categorize feedback into local feedback and global feedback according to feedback focus.

Feedback scope is defined as the extent to which teachers should provide their students with feedback (Lee et al., 2015; Lee, 2017). That is, this theme is concerned with whether teachers should offer feedback targeting a range of errors (comprehensive feedback) or limited types of errors (focused feedback). Currently, many researchers have justified value of focused feedback. Theoretically, focused feedback is friendlier to L2 learners, since such a practice can help them avoid being cognitively overloaded, which makes them have extra cognitive resources to deal with new input effectively (Sheen et al., 2009; Cheng and Zhang, 2021b; Zhang and Cheng, 2021). Empirically, they have verified the effectiveness of such WCF on enhancing L2 learners' writing accuracy in target linguistic structure(s) (Bitchener and Knoch, 2009, 2010; Li and Roshan, 2019).

However, because of the lack of ecological validity, focused feedback is questioned by some L2 researchers (e.g., Ferris, 2010; Storch, 2010; Van Beuningen, 2010; Karim and Nassaji, 2020; Lee, 2020; Zhang T. F., 2021). According to their views, focused feedback does not align with the normal practice in L2 writing contexts, where teachers tend to correct a number of errors. Furthermore, teachers undertake the responsibility to help their students produce high-quality writing (Bitchener and Ferris, 2012). In this sense, it is not sufficient for teachers to adopt a focused approach to mark students' writing. Several studies have explored the efficacy of comprehensive feedback, finding that it can facilitate overall writing accuracy in revised texts/new pieces of writing (Van Beuningen et al., 2012; Bonilla López et al., 2018).

Aside from feedback scope, teachers are also concerned with the strategies that should be used to deliver their feedback. Generally, two broad categories of feedback exist with regard to strategies: Direct and indirect feedback. Direct feedback is known as teachers offering answers to errors directly, while indirect feedback means that teachers only identify and indicate errors for their students without corrections (Lee, 2017; Cheng and Zhang, 2021a; Zhang and Cheng, 2021). Until now, the relative effectiveness of direct and indirect written feedback is inconclusive due to the variations of participants' language proficiency. However, many scholars have claimed that direct feedback is more beneficial for L2 learners, particularly low-proficiency ones.

The last theme is feedback orientation, which includes two sub-themes: Positive and negative feedback. The former refers to comments affirming that students' writing has met a standard such as “good grammar”, “clear organization”, and “the task is well achieved”. In contrast, negative feedback is defined as teachers' comments, indicating that there are some errors, problems or weaknesses in students' writing (Hyland and Hyland, 2001). The two types of feedback, positive and negative, play different roles. While positive feedback contributes to students' motivation and self-esteem, negative feedback can enable students to be aware of their problems and weaknesses even though it may affect students' emotion negatively, making them diffident and discouraged (Connors and Lunsford, 1993; Ferris, 1997; Ashtarian and Weisi, 2016).

### Language Teachers' Beliefs

With the rise of cognitive psychology, teacher beliefs have been established as an important issue in the area of education (Pajares, 1992; Fang, 1996; Sun and Zhang, 2021). This research agenda advocates that researchers should take teachers' mental lives into consideration and teachers' observable practices within their teaching contexts should be linked to their thinking (Borg, 2003, 2015). In the existing literature on teacher belief, researchers have paid much attention to the definition of belief and factors contributing to teacher belief (Gao and Zhang, 2020).

Prior to conducting research on teacher beliefs, researchers should present an accurate definition about beliefs. However, it is not an easy task, since belief is a “messy construct” (Pajares, 1992, p. 302; see also Fang, 1996), which is difficult to conceptualize. The main reason for the difficulty is that belief is a complex concept with many interchangeable terms and these terms share the main characteristics of belief, which lead to “definitional confusion” (Borg, 2003, p. 83). Currently, approximately 60 distinctive terms have been employed to refer to belief (Borg, 2015, 2019). Examples of such alternative terms include “opinion”, “attitude”, “theory”, “maxim”, “conception”, and “principle”. Although there is a proliferation of terms in literature, most represent a similar concept. As Woods (1996) stated, the various terms do not mean that scholars research conceptually different things. Such interchangeable terms, to a large extent, result in the difficulty in examining teacher belief.

According to the above discussion, the operational definition of teacher belief in our study is “the unobservable cognitive dimensions of teaching—what teachers know, believe, and think” (Borg, 2003, p. 81; see also Borg, 2019). In other words, teacher belief includes a series of assumptions, values, feelings, and attitudes toward teaching (Borg, 2019).

In general, teacher belief is not formed overnight. Instead, teachers develop their teaching beliefs throughout their whole professional life (Johnson, 1994; Flores, 2001). Based on Borg (2015), there are four factors influencing teacher belief, which include schooling, professional coursework, classroom practice, and contextual factors.

Schooling refers to teachers' previous learning experiences as students in school. Prior learning experiences are pivotal in the formation of teachers' beliefs and considered the most influential factor contributing to their beliefs (Borg, 2015). Through “apprenticeship of observation” (Lortie, 1975), teachers shape their beliefs regarding teaching and learning. Professional coursework, known as teacher education or teacher training, is recognized as another source of teacher belief. Commonly, teachers have opportunities to receive teacher education to enrich their pedagogical knowledge and develop their professional skills, which may influence their existing beliefs, or enable them to reframe their beliefs about teaching. However, compared with prior schooling, professional coursework exerts a relatively moderate influence on teacher belief. When it comes to classroom practices, it is known that teacher belief guides and informs their classroom practices. However, classroom practices, in turn, influence the formation of teacher belief (Phipps and Borg, 2009). Specifically, teachers may assess their pedagogical contexts, including teaching syllabus, workload, students' proficiency and needs, and school policies, as well as evaluate their actual teaching practices against students' learning outcomes to improve their teaching efficacy, which may modify or strengthen their beliefs (Borg, 2015). Contextual factors mediate and influence the relationships between teachers' beliefs and pedagogical practices (Rahimi and Zhang, 2015; Bao et al., 2016; Sun and Zhang, 2021). It is common that teachers' teaching practices are not always in line with their beliefs. Such belief-practice mismatches are probably attributed to contextual factors such as school policies, teachers' teaching workload, and examinations (Gao and Zhang, 2020; Yu et al., 2020).

### L2 Teachers' Written Feedback Beliefs

As noted earlier, the extant studies regarding teacher written feedback mainly focus on the WCF effectiveness, but teachers' beliefs about written feedback remain under-explored (Evans et al., 2010; Mao and Crosthwaite, 2019). To examine such an issue, Lee (2009) conducted a study to investigate NNES secondary EFL teachers' beliefs about written feedback in the Hong Kong. She found that teachers espoused a series of beliefs regarding written feedback. For example, they believed that teachers should not focus only on language and they should implement focused feedback. Similarly, Alkhatib (2015) adopted a case study to examine NNES teachers' written feedback beliefs in a Saudi Arabic tertiary EFL context. His study reported that these teachers shared similar beliefs in terms of feedback scope and strategy. Specifically, they believed in comprehensive and direct feedback. However, such teachers differed in their beliefs regarding feedback focus. In addition to EFL teachers, Junqueira and Payant (2015) examined how a novice NES ESL teacher conceptualized written feedback, which revealed that the teacher favored global feedback in response to L2 learners' written assignments.

Few studies have examined such an issue in mainland China (e.g., Yang and Gao, 2012; Mao and Crosthwaite, 2019; Wei and Cao, 2020). For example, Mao and Crosthwaite (2019) examined how five tertiary NNES EFL teachers theorize written feedback in a mainland Chinese EFL setting. Gathering data from semi-structured interview and questionnaire, they reported that teachers believed in focused and direct feedback. They also held that teachers should attach more importance to global issues than local ones. These studies add our knowledge regarding teachers' beliefs about written feedback in mainland China; yet they do not take the NES EFL teachers' beliefs into consideration.

After the review of the above studies, several gaps can be summarized. Firstly, scant attention has been paid to L2 teachers' beliefs regarding written feedback in the mainland China. Furthermore, the existing literature in this line does not examine teachers' beliefs about written feedback systematically. Specifically, they have mainly investigated teachers' beliefs regarding feedback scope, strategy, and focus. Thus, much remains to be known about L2 teachers' conceptions related to other dimensions of written feedback. More importantly, the extant studies tend to focus on NES/NNES L2 teachers' written feedback beliefs. Such studies fail to paint a comprehensive picture, since they do not take into account teachers' L1 backgrounds. Given the impact of teachers' sociocultural and language backgrounds on their beliefs (Lee, 2017; Borg, 2019; Hyland and Hyland, 2019), the comparative studies on NES and NNES teachers' beliefs regarding written feedback are called for. To bridge the gaps, our study intended to address the following research question:

*What are NES and NNES teachers' beliefs regarding written feedback in the mainland Chinese EFL context?*

## Method

### Context and Participants

Before conducting our study, we obtained the ethics approval from the Ethics Committee on Human Participants of The University of Auckland, New Zealand. Participants were told the aims, procedures, their rights, and possible benefits they would gain from our study through participant information sheets. They were also ensured that we would try our best to protect their identity. Our study was implemented in a central province of mainland China, and we selected the potential participants through a purposive sampling strategy. The employment of such a strategy was due to its strength that it can help researchers select the participants “who can provide rich and varied insights into the phenomenon under investigation so as to maximize what we can learn” (Dörnyei, 2007, p. 126).

To approach the possible participants, we contacted the Deans from three universities and explained our study for them carefully. With their permission, we distributed the participant information sheets and consent forms to all the EFL teachers in each University through the Dean's secretary. Finally, four NES and four NNES teachers agreed to participate in our study and submitted consent forms. Table 1 shows their demographic backgrounds.

**Table 1:** Participating teachers' profile.

**Name (pseudonym)**	**NES/NNES**	**Country of origin**	**EFL writing teaching experience**	**Academic qualification**	**Major**
Jason	NES	US	4 years	Bachelor	Linguistics
George	NES	US	3 years	Master	TESOL
Bruce	NES	US	6 years	Master	Literature
Christine	NES	US	4 years	Bachelor	Literature
Yan	NNES	China	8 years	Master	TESOL
Juan	NNES	China	5 years	Master	Literature
Han	NNES	China	3 years	Master	Applied linguistics
Qin	NNES	China	4 years	Master	Linguistics and applied linguistics

During the period of data collection, all the participants in the three universities offered *English Writing Course* for second-year students in English major, who can be regarded as the intermediate EFL learners. These students learned the same writing course through a similar curriculum. Such a writing course was delivered once a week in a 16-week semester, which was expected to foster and enhance students' writing ability and help them pass TEM-4^1^. In this course, students were asked to complete four or five writing tasks in/after class. Teachers rated the writing samples based on TEM-4 writing rubrics and gave written feedback, after which students were asked to revise their written texts based on teachers' written feedback.

### Data Collection

To address the research question, we collected data mainly through semi-structured interviews. Interview, as an important research instrument, is widely used in qualitative studies to explore participants' opinions and views about the phenomenon under investigation, which cannot be observed directly (Creswell and Creswell, 2018). As such, interview is a popular and effective research tool to examine teachers' beliefs, for they reside in teachers' mind and are unobservable thought process (Borg, 2003, 2015). There are three types of interviews: Unstructured, semi-structured, and structured. The utilization of semi-structured interviews in our study was based on our consideration of its advantages. Such a form of interview enables interviewers to guide the direction of research without diverting through preparing some guiding interview questions in advance and also provides interviewees with sufficient flexibility to elaborate on their views (Dörnyei, 2007).

Each participating teacher was contacted to arrange the time and place, convenient for the interview. Before each interview, we had a chat with each teacher to create a relaxing atmosphere and establish a good rapport. The interviews with the eight teachers were conducted individually using the prepared questions (see Appendix A), and each interview lasted 45–60 min. The interviews with NES teachers were implemented in English and those with NNES teachers in Chinese. The interviews were audio-recorded for analysis with the participants' permission. Out of the ethical considerations, we reassured the participants that their identities and information would be kept confidential and that we would use pseudonyms when reporting the findings. In doing so, such participants would able to express their thoughts and opinions without anxiety (Zhang, 2010).

In addition to interviews, we also collected some documents including teachers' teaching plans and power points, writing course syllabus, and TEM-4 writing rubrics. Such documents could enrich our understanding of these participants' written feedback beliefs and provide contextual information for our study.

### Data Analysis

In this study, all the interviews were fully transcribed. To ensure the reliability, all the interview transcripts were sent to the eight teachers for member checking. Given that the interviews with NNES teachers were conducted in Chinese, the transcripts were analyzed in the original language and we translated them into English when they were needed for reporting the findings. Such a decision was that translation may lead to the loss of information due to the difficulty in finding the equivalent words, phrases, and concepts in the source language and target language (Gao and Zhang, 2020). Once the transcriptions were completed, the analysis was done manually.

Firstly, we read the interview transcripts several times to have a general picture of the data. The repeated reading enabled us to be more familiar with the data and obtain a general understanding of data (Marshall and Rossman, 2016; Creswell and Creswell, 2018). We also made some reflective notes and recorded some concepts, ideas, and thoughts in the margins of the transcripts as they occurred to us when he read and re-read the data.

Next, we used the thematic analysis to code teachers' beliefs. Thematic analysis is an effective approach to processing qualitative data, as it identifies, analyzes, and interprets the themes within the dataset (Braun and Clarke, 2006). Thematic analysis can be implemented by either inductive or deductive coding approaches. For this study, a deductive approach was used as the main strategy, as it could tell us what is known and what is unknown about the phenomenon being examined (Patton, 1986). Four themes were emerged and identified: Feedback scope, feedback focus, feedback strategy, and feedback orientation.

We coded the transcripts line by line and noted a code in the right-handed margins of transcriptions. The words, phrases, and sentences produced by the teachers relevant to the four themes were marked. To ensure the reliability of the research findings, we invited an EFL writing teacher with her master's degree in applied linguistics, who did not participate in our study as a co-coder. Approximately 20% of the recording transcripts were selected randomly and coded by us independently. Any disagreements were discussed until they were resolved, after which the first author coded all the remaining data.

## Findings

### Feedback About Feedback Purpose

All the participants acknowledged the importance and value of written feedback, stating that it was writing teachers' responsibility to offer feedback to their students and that it played an irreplaceable role in L2 writing classrooms. However, when interviewed, the eight teachers expressed different purposes for giving feedback on their students' writing, which are elaborated as follows.

Most teachers (six out of eight) explained that they offered students written feedback to improve their writing performance. They agreed that teachers' feedback enabled students to recognize the errors and problems and avoid them in the subsequent writing tasks. For example, Bruce responded in the interview:

*Bruce: With feedback, students had a deep insight into their errors and problems. They were unlikely to repeat them in the follow-up writing tasks, which contributed to their writing proficiency*.

Bruce emphasized the importance of feedback in improving students' writing proficiency. Likewise, Qin reported that feedback made students aware of the areas to which they should pay attention and provided directions for further improvement, with which students were likely to produce better written products.

From another perspective, three teachers regarded teacher feedback as a useful instrument to inform their pedagogical practices in writing instruction. In their opinion, providing feedback had important implications for their actual teaching. When asked whether teachers should give feedback to students' writing, Christine replied:

*Christine: Teacher feedback not only played an important role in students' writing learning, but also in teachers' instruction. Guided specifically by the feedback given to students, teachers could know the areas which they should emphasize and those that they could omit in the follow-up teaching process. Thus, teachers' teaching could be more effective and efficient*.

Juan responded similarly that teachers' pedagogical practices benefited considerably from the opportunity to provide feedback. In stating her view, she pointed out that feedback provision prompted teachers to understand whether their teaching was effective and helpful in students' writing development. She further added that such a practice could inform teachers' adjustment of their teaching content.

### Beliefs About Feedback Scope

According to the interview data, three out of four NES teachers (George, Jason, and Christine) voiced that teachers should provide students with focused written feedback, while Bruce argued that comprehensive feedback was more suitable. Their points are discussed in the successive paragraphs.

The three teachers, who supported focused feedback, stated such feedback was important in L2 writing and it was unnecessary for teachers to correct a wide array of errors and problems in students' writing. For instance, Jason explained that focused written feedback could reduce students' burden in revision and make it possible for them to have a deep insight into the specific errors they made. He further added that when he was a student, his teachers tended to focus on two or three types of errors in providing feedback, and it was, he supposed, a better approach to feedback provision.

George's belief in focused feedback was based on students' needs. As he remarked in the interview:

*George: As far as I am concerned, teachers' feedback affects Chinese EFL learners' emotions greatly, in particular low-achieving learners'. Too many corrections in red ink discourage them. To cultivate their confidence and enhance their motivation in English writing, it is more reasonable for teachers to provide feedback in a focused way*.

Christine also upheld focused feedback, but for a different reason. She noted that compared with comprehensive feedback, focused feedback was time and energy saving. Thus, it was a more practical approach in the Chinese EFL context, in which teachers are confronted with heavy workloads and are responsible for large-size classes. Here is what she reported:

*Christine: I think focused feedback is a better approach to feedback provision because of heavy workloads and large-size classes. As for me, I need to be responsible for another three courses in this semester and there are more than 30 students in my writing class, so it is really a tough task for me to provide each student with comprehensive feedback*.

In contrast, Bruce preferred comprehensive feedback. In the interview, he explained that teachers need to correct all or most errors and problems in students' writing. Although providing such feedback was time and energy consuming, it was a sense of responsibility that encouraged him to do so.

Compared with NES teachers, most NNES teachers agreed that teachers should offer comprehensive feedback to students. Specifically, Juan, Yan, and Qin supported the use of comprehensive feedback, but they had different reasons for their beliefs. For instance, when interviewed whether teachers should highlight different types of errors for students, Juan and Yan expressed, “Comprehensive feedback should be used in practice in that it is more helpful in improving students' general writing proficiency, which is the ultimate goal of our writing instruction.” In their opinions, teachers needed to help students enhance overall writing proficiency, not just performance in some specific areas of writing. They believed that comprehensive feedback was a suitable approach to achieve such a goal.

Unlike Juan's and Yan's reasons, Qin's justification was that if teachers provide feedback selectively, uncorrected errors may appear in the follow-up writing. In this sense, students might repeat some types of errors constantly in their writing.

*Qin: I believed that comprehensive feedback is more beneficial to students' learning because it can prevent the fossilization of errors. In reality, many Chinese students are not capable of identifying and correcting errors by themselves. If teachers leave some errors unmarked, students may make the errors constantly in the subsequent writing tasks*.

Differently, Han opined that focused feedback was a better approach to provide feedback. In explaining his view, he presented two reasons. One was that focused feedback was time and energy saving, aligning with Christine's opinion. More importantly, he argued that focused feedback was of great benefit for L2 learners' writing development, as they had opportunities to detect and correct unmarked errors independently, which was very important for their writing development in the long run.

### Beliefs About Feedback Strategy

Overall, NES teachers' beliefs about feedback strategies varied. George and Jason agreed that it is necessary to mix both direct and indirect feedback strategies but gave different reasons for their beliefs. For example, in the interview, George replied, “The best way is to combine the direct and indirect feedback, which can maximize the effectiveness of teacher written feedback.” In the interview, he advocated the concurrent use of direct and indirect feedback due to their individual advantages. Direct feedback, he asserted, enabled students to understand the correct forms of their errors instantly. In contrast, indirect feedback could save teachers' time, which could improve the efficiency of feedback provision. As he believed, to increase students' awareness of errors and improve teachers' efficiency of providing feedback, the integration of both direct and indirect feedback was an optimal strategy.

Like George, Jason supported the belief that teachers should combine direct and indirect feedback in L2 writing classrooms. In the interview, he also mentioned the individual merits of direct and indirect feedback strategies, but the major reason for his belief was that it could best meet students' needs. From his perspective, in Chinese EFL classrooms, students had various levels of English proficiency and teachers' feedback should be different. To specify, students with low English proficiency should be provided with feedback directly, while those who were advanced EFL learners should be given indirect feedback:

*Jason: In the Chinese EFL writing classrooms, students' English proficiency varies. For students with high English proficiency, they need to foster self-editing ability. Thus, indirect feedback is more suitable. For low-achieving students, direct feedback is supposed to be offered, by which they can understand how to correct errors*.

Jason took students' differing needs into consideration in the formation of his belief regarding feedback strategies. As he stated, to foster the ability to correct errors independently, teachers should provide advanced English learners with indirect feedback, while direct feedback should be given to low-achieving ones to facilitate their understanding of the correct forms of their errors. In this way, the needs of students with different levels of English proficiency could be satisfied.

The other two NES teachers held opposing beliefs about feedback strategies. Bruce was in favor of direct feedback, claiming that it was teachers' responsibility that made him feel obliged to provide students with direct feedback, as illustrated by his words in the interview:

*Bruce: We (teachers) have the obligation to locate errors and present the correct answers for students. This is our job. If we do not do like this, we will not fulfill our responsibility because many students, particularly those with low English proficiency probably do not know how to correct errors*.

In contrast, Christine was supportive of indirect feedback strategy, offering two justifications for her belief. Firstly, she argued that it was not practical to offer direct corrections to errors on each student's writing in the Chinese EFL context, in which the classes were large and teachers had tight working schedules. In this situation, providing direct feedback took an amount of teachers' time and energy. More importantly, she added that indirect feedback enabled students to become personally engaged with error correction more profoundly. Thus, they had a better understanding of their errors, which benefited their long-term development in writing. Based on the two reasons, she believed in indirect feedback.

Similar to NES teachers, the four NNES teachers' beliefs regarding feedback strategies did not reach an agreement. Juan and Qin agreed that teachers should present their written feedback directly to students. Juan put emphasis on the advantages of direct feedback. Firstly, it could focus students' attention on their errors and problems in writing, whereas if the errors were just located, students may not take them seriously. Secondly, direct feedback prompted students to realize the correct forms immediately.

*Juan: Providing students with direct corrections should be encouraged. If teachers just indicate errors by underlines, students may not pay enough attention to them. Besides, direct feedback contributes to students' immediate understanding of error correction*.

Qin's belief in direct feedback was due to her teaching experience. After several years of teaching, she found that indirect feedback was not very effective, as many students found it difficult to correct errors when she gave indirect feedback. To facilitate students' error correction, she abandoned it and employed a direct feedback strategy.

By contrast, Yan espoused the use of indirect feedback, citing an old Chinese saying “授人以鱼不如授人以渔 (It is much better to teach somebody to fish than give somebody a fish)”. According to her explanations, although it is less challenging for students to understand direct than indirect feedback, indirect feedback enabled students to engage with teacher feedback more deeply. She emphasized that this engagement could improve their self-editing ability, which contributed to greater progress in L2 learners' writing in the long run. Yan, therefore, favored the employment of indirect feedback in feedback provision because it enabled students to gain a deeper insight into their errors and fostered their self-revising skills.

Unlike the above three teachers, Han believed in the concurrent use of direct and indirect strategies while providing written feedback. He thought that teachers should treat different types of errors with different feedback strategies.

*Han: When the problems are related to content and organization, I tend to use indirect feedback. In other words, I only indicate the problems. In contrast, in terms of errors in grammar and vocabulary, I prefer to give corrections directly*.

### Beliefs About Feedback Focus

As for the focus of feedback, the four NES teachers unanimously agreed that L2 teachers should give more importance to problems in content and organization and provide students with feedback focusing on global issues. The details of their beliefs are presented in the following paragraphs.

When asked for beliefs regarding feedback focus, George articulated that teachers, because of their role, should give priority to issues related to content and organization. Writing teachers' main responsibility, rather than improve students' writing accuracy, was to help students develop their ideas clearly and adequately, be aware of the global and local structures of their writing and pay attention to the logical relationships between sentences. If they paid too much attention to grammatical accuracy, they would be grammar teachers instead of writing teachers. It was clear that George believed in global feedback and that his belief was ascribed to writing teachers' identity. Bruce, like George, emphasized writing teachers' identity as well. As he responded in the interview:

*Bruce: I think we (writing teachers) should always remind ourselves that we are writing teachers. As writing teachers, we need to address the problems in global areas instead of focusing on the use of grammar. So, we assume the responsibility to teach students how to compose a good text, which contains relevant and clear content as well as well-organized structure, not just error-free sentences*.

The same belief in global feedback was apparent in Jason's interview, but for a different reason. In his view, it was the teaching objectives that contributed to his belief about feedback focus, claiming that writing teachers should take the goals stipulated by writing course into consideration when providing feedback. For example, the English writing course aimed mainly to foster students' genre awareness and teach them how to develop their ideas reasonably, as well as structure their ideas appropriately, not just focus on accuracy. Guided by such objectives, he deemed that teachers should pay more attention to problems in content and organization when giving feedback to students.

Christine said she believed that teachers should stress problems at global levels, because of the nature of writing. She explained that a piece of good writing was more than a cluster of error-free sentences. As she responded:

*Christine: We should understand the nature of writing. Its nature is to convey an author's ideas to readers, and writing serves as a media to communicate with others. Therefore, it involves more than grammar and vocabulary. In other words, even though a student writes an essay with few errors in language, it also makes no sense if it is irrelevant to the topic and structured in a messy organization*.

The excerpt indicates that Christine espoused the provision of feedback on global issues in that she realized that the purpose of writing was to communicate with readers rather than create a collection of error-free sentences. She thought teachers should be concerned more with global issues and should not overemphasize writing accuracy.

Interestingly, the majority of NNES teachers also believed in the emphasis on content and organization in giving feedback. For example, in the interview, Yan replied that content and organization deserved writing teachers' attention most. In her response, she suggested that students might have difficulty identifying and remedying problems with content and organization by themselves.

*Yan: English writing course is open for English major students, so they have some knowledge of grammar and have the ability to correct grammatical errors by themselves. However, it is very difficult for them to detect global issues such as clear and convincing ideas, cohesion, and coherence, let alone solve them*.

Consistent with Yan, Han and Qin favored the provision of global feedback in their belief systems. Both of them stressed the variations between Chinese and English writing rhetoric in global areas. As Han reported in the interview, “As two different languages, Chinese and English have many differences in writing conventions in organization. For example, in Chinese writing, a lot of supporting details are presented before proposing ideas. In contrast, in English writing, a topic sentence needs to be formulated at the beginning of each body paragraph, followed by supporting details.” Such differences, he believed, may result in students having problems with global dimensions of English writing, which suggested that teachers should prioritize such problems when providing feedback.

Different from other teachers, Juan said she believed that linguistic errors should be given priority to when L2 writing teachers give feedback. As she explained in the interview:

*Juan: Grammar and vocabulary are the foundations of English writing. Even if a student produces an essay without cohesion, outstanding ideas, or reasonable organization, it can be understood if he/she uses appropriate words and writes error-free sentences*.

She continued by making an analogy. As she put, language was to writing what clothes were to people. If a person wore dirty clothes, other people would be unhappy. In a similar vein, if there were many grammatical errors in writing, other people would lose interest in reading it. Therefore, she attached great importance to feedback on language.

### Beliefs About Feedback Orientation

When interviewed whether writing teachers should provide positive or negative comments on students' writing, two of the four NES teachers remarked that teachers should give positive comments, while the other two NES teachers had differing beliefs.

George and Christine both responded that positive feedback should be provided, pointing out that identifying the strengths of students' writing could boost their confidence and promote their motivation in writing. They continued by arguing that nobody would be happy and feel motivated if his/her writing was full of comments indicating weaknesses and problems. As Christine noted in the interview:

*Christine: As for me, it is necessary for teachers to use written comments to highlight students' strengths when providing feedback. I remember that when I was a student, my writing teachers often did so. Such positive comments enhanced my confidence and interest in writing and encouraged me to do better*.

Christine attributed her strong advocacy for providing positive comments on students' writing to her previous schooling experiences, which benefited her learning in writing greatly.

However, Bruce said he believed that negative comments should be provided in practice. In the interview, he emphasized the need to give negative feedback, so that students could understand their weaknesses and problems in writing; he justified his belief as follows:

*Bruce: I believe negative feedback which points out students' weaknesses and problems is more beneficial for them because they pay more attention to negative comments. Additionally, pointing out weaknesses is more meaningful than providing some empty praise like “good” or “well done” in that it (empty praise) may not stimulate students' reflections*.

Jason showed a preference for using negative and positive comments concurrently in feedback provision, saying he thought giving feedback should take students' English proficiency into consideration. He went on to explain that advanced English learners needed more negative feedback so that they could understand their problems and weaknesses clearly, thus enabling them to make greater progress in writing. However, for students with low English proficiency, teachers should highlight the strengths of any aspects of their writing, acknowledge their performance, and boost their writing confidence so they would be more willing to make effort. Jason's attitude toward the provision of positive and negative comments took into account students' English proficiency to meet their needs.

Surprisingly, three out of four NNES teachers (Juan, Yan, and Qin) supported giving negative feedback to highlight students' problems in writing, contending that it was less likely for students to improve if they did not understand their inadequacies or problems. Negative comments helped raise awareness of their weak areas, and therefore do better in writing. As Juan and Yan remarked in the interview:

*Juan: It is important to give feedback comments to indicate students' problems because such comments are what students need and can really benefit them. If teachers do not do so, students will not pay due attention to their writing problems or even they will not be aware of their weaknesses in writing at all*.

*Yan: I think that the positive comments such as “good points”, “good conclusion” or “well organized” are meaningless because with such comments, students do not reflect on their writing. As teachers, we should assume the responsibility to identify and diagnose students' problems in their task performance rather than compliment them*.

The above excerpts show that these teachers emphasized the role of negative comments in feedback provision. Whereas Juan valued negative comments to help students understand the directions for further improvement, Yan thought that teachers' responsibility was to facilitate students' understanding of their problems in greater depth.

In contrast, Han supported the combination of positive and negative comments due to the respective advantages of positive and negative feedback. He explained that positive feedback, affirming one or several dimensions of students' writing met standards, could encourage and motivate students, while negative feedback drew students' attention to their writing problems. To balance enhancing students' confidence and raising their awareness of their inadequacies in writing, combining positive and negative feedback was the ideal approach. As neither positive nor negative feedback alone was better, it was necessary and more effective to include both strengths and weaknesses in feedback provision.

## Discussion

### Beliefs About the Purpose of Feedback

The interviews showed that the teachers recognized the value of written feedback, endorsing teachers giving students feedback on their writing. Teachers' acknowledgment of the significance of feedback is consistent with the prior literature in the fields of both written feedback (Diab, 2005; Evans et al., 2010) and oral feedback (Roothooft, 2014; Bao, 2019; Kartchava et al., 2020).

The participants in this study presented two purposes for giving feedback to students. The major purpose was to promote students' writing performance, which was not surprising. As indicated by Schmidt (1990), feedback enables learners to notice their output deficits and realize the gap between what they can produce and what they need to produce, thereby contributing to their writing performance. The positive efficacy of teacher written feedback on L2 writing have been supported by a great many (quasi-) experimental studies (Bitchener and Knoch, 2009, 2010; Shintani and Aubrey, 2016; Zhang T. F., 2021). As Bitchener and Stroch (Bitchener and Storch, 2016) concluded, written feedback can facilitate L2 learners' writing accuracy, even if one-off written feedback is offered.

The second purpose for teachers' feedback provision was to inform their pedagogical practices. This purpose of feedback provision can also be seen in previous studies (e.g., Alkhatib, 2015). After feedback provision, teachers can collect information about “what students understand and what they do not understand” (Hattie and Timperley, 2007, p. 90). With such information, teachers can adjust their instructional content and focus in the subsequent teaching to enhance their teaching efficacy.

### Beliefs About Feedback Scope

Our study revealed that the majority of NES EFL writing teachers favored focused feedback, advocating that teachers should correct a few types of errors and leave other uncorrected. This aligns with the findings of prior literature (Mao and Crosthwaite, 2019), in which L2 writing teachers showed a preference for focused feedback. Such a finding is also consistent with intervention studies, investigating the efficacy of focused feedback (Shintani and Ellis, 2013). All these investigations have documented the positive effects of focused feedback on improving L2 learners' accuracy in target structure(s).

In contrast, three out of four NNES teachers believed in comprehensive feedback. Such a finding agrees with the findings reported by prior research (e.g., Lee, 2011; Alshahrani and Storch, 2014; Alkhatib, 2015). For example, conducting a case study, Alshahrani and Storch (2014) reported that tertiary EFL writing teachers espoused comprehensive feedback in Saudi Arab. Similarly, Lee (2011)'s case study found that teachers favored feedback targeting a variety of errors in the Hong Kong secondary EFL contexts. Furthermore, NNES teachers rationalized their beliefs in comprehensive feedback such as avoiding the fossilization of errors and enhancing L2 students' overall writing performance. This corresponds to Van Beuningen et al. (2008, 2012), whose quasi-experimental studies justify the importance of comprehensive feedback. In their studies, comprehensive feedback enhanced L2 learners' general writing accuracy rather than accuracy in the limited pre-selected linguistic feature(s).

In our study, there was an obvious discrepancy between NES and NNES teachers' beliefs with regard to feedback scope. Their contrasting beliefs may be attributed to two reasons. One is their previous learning experiences, which is regarded as an important source of teacher belief (Borg, 2015). Specifically, in EFL contexts, as comprehensive feedback is a ubiquitous practice adopted by teachers in feedback provision (Furneaux et al., 2007; Lee, 2008, 2011), the NNES teachers probably received comprehensive feedback from their teachers when they were students at school. Due to the apprenticeship of observation (Lortie, 1975), they were very likely to believe in comprehensive feedback. In comparison, for NES teachers, they tended to have high levels of English proficiency with few errors in their writing, their teachers may have used a focused approach to provide feedback. This perhaps contributed to their advocacy for focused feedback. Jason, for example, remarked in the interview that his teachers often employed a focused approach in feedback provision when he was a student.

The other plausible reason for such a discrepancy was the influence of different educational systems. In the Chinese educational schema, deeply rooted in Chinese traditional culture, as ancient scholar Han Yu writes in his *On Teachers*, a teacher is someone who is capable of propagating doctrines, imparting knowledge, and resolving doubts. In this sense, teachers are required to correct students' errors extensively to reduce their anxiety and ensure their full development in learning; they will be considered irresponsible and lazy if they overlook their students' errors. Born and brought up in such an entrenched value, NNES teachers probably believe it is their duty to give feedback targeting a variety of errors. In contrast, western education system gives more importance to students' agency in learning and underplays teachers' role (Rao and Li, 2017; Yan et al., 2021). NES teachers who were brought up and educated in this culture may support that it is important for students to accept the responsibility to identify their errors by themselves, and so it is unnecessary for teachers to mark errors comprehensively.

### Beliefs About Feedback Focus

Interestingly, teachers in our study shared a belief about feedback focus, regardless of their L1. That is, they reached a consensus that teachers should pay more attention to global dimensions, that is, problems in relation to content and organization, in providing students with written feedback. Such a finding can be seen in many studies (Montgomery and Baker, 2007; Junqueira and Payant, 2015; Zhao, 2019).

More interestingly, although the two groups of teachers believed that teachers should give more importance to global issues in feedback provision, they had different reasons for their beliefs. NES teachers proposed three explanations. Firstly, teachers attributed their beliefs to their identity as writing teachers. This holds true for George and Bruce, who regarded themselves as writing teachers rather than grammar teachers. Hence, they upheld global feedback in their belief systems. The second reason was the teaching objectives of English writing. The aims of writing instruction, as Jason argued, were to make students understand how to develop their ideas more effectively and how to organize their writing in an appropriate structure. Influenced by these objectives, it was necessary for English writing teachers to provide more feedback on problems in content and organization. This aligns with Wang (2015) study, where English writing teachers conceptualized English writing instruction as teaching students to develop and structure ideas instead of just focusing on grammatical accuracy. The final reason given was the nature of writing. According to Christine, writing was by no means a cluster of error-free sentences; it was more about the communication between authors and readers. She claimed that writing teachers should be concerned with ideas and structure of writing when providing feedback. The view that writing quality is more than accuracy in language is evident in Zhao (2019) investigation, in which Chinese EFL writing teachers ranked criteria for judging writing in the order of importance as organization, content, and language (grammar and vocabulary).

In comparison, one of the two reasons proposed by NNES teachers for their beliefs in global feedback was students' needs. L2 learners tend to find it difficult to identify and solve global issues on their own, as they fail to have adequate English writing rhetorical knowledge (Hinkel, 2002). As Yan argued, it was challenging and demanding for Chinese tertiary EFL learners to detect and remedy problems in global areas independently, and that teachers needed to assist them in such dimensions of writing through feedback. The other reason was the difference between Chinese and English writing rhetoric, as documented by researchers in the realm of intercultural rhetoric (e.g., Kaplan, 1966). As NNES teachers had Chinese and English at their disposal, they perceived such differences. To help students avoid the negative transfer from Chinese writing rhetoric, Han and Qin opined that global issues should be emphasized through feedback. Loi and Evans (2010) also claimed that different rhetoric patterns in Chinese and English writing may pose obstacles and challenges to Chinese EFL learners, who are used to Chinese rhetoric patterns while composing their English writing.

### Beliefs About Feedback Strategy

When it comes to feedback strategy, our study revealed that there was no consensus in teachers' beliefs on how to deliver written feedback, both within and across groups. Teachers' beliefs about feedback strategies were divergent, which are in line with the fact that the differential effectiveness of direct and indirect feedback was inconclusive.

For the convenience of discussion, NES and NNES teachers here are put together. Three teachers (Bruce, Juan, and Qin), in our study, considered that students should be given direct answers, a view that has been reported in existing literature (e.g., Mao and Crosthwaite, 2019). However, considering that the provision of direct feedback may lead to students' dependence on their teachers (Lee, 2008), teachers' beliefs in direct feedback appear to indicate that they are in favor of teacher-led feedback and their students probably do not undertake the responsibility to correct errors independently. Despite different reasons provided by the teachers to rationalize their beliefs, their strong orientation toward direct feedback may be related to their sense of responsibility. That is, teachers perhaps regarded correcting students' errors explicitly as their job. As Bruce said in the interview, if he did not correct students' errors directly, he would be feel guilt. Similar comments are also found in prior literature (e.g., Alkhatib, 2015; Mao and Crosthwaite, 2019). As Bitchener and Ferris (2012) stated, written feedback is regarded as a part of teachers' overall responsibility. Thus, to fulfill their responsibility better and enhance their professional identity (Kroll, 1990), teachers may believe in direct feedback.

Among the eight teachers, two teachers (Yan and Christine) showed a preference for indirect feedback, which is close to Lee (2009) investigation, where EFL teachers preferred indirect to direct feedback in practice. In our study, the two teachers claimed that students benefited more from indirect feedback, as it enabled them to engage with error correction deeply and fostered their self-revision ability. This would facilitate students' long-term writing development. As Bitchener and Knoch (2008) argued, indirect feedback “requires students to engage in guided learning and problem solving and, as a result, promotes the type of reflection, noticing and attention that is more likely to foster long-term acquisition” (p. 415). Teachers' beliefs in indirect feedback are probably associated with the contextual factors in EFL contexts (i.e., heavy workloads and large-size classes). This is particular true for Christine. In the interview, she complained about heavy workloads and large-size classes in the Chinese EFL context. As previous studies found, Chinese EFL teachers tended to be faced with these two constraints (Gao, 2018; Mao and Crosthwaite, 2019). In such a context, it is overwhelming for teachers to provide all students with direct corrections due to time and energy consumption. Thus, indirect feedback seems to be a suitable strategy.

The rest of teachers, George, Jason, and Han supported a mixture of direct and indirect strategies for feedback. Their beliefs are based on two factors. One is students' needs. As Jason stated, students in Chinese EFL writing classrooms varied in their English proficiency and should be treated differently to meet their different needs. In the existing literature, researchers encourage teachers to take students' needs such as proficiency and motivation into consideration when deciding on feedback strategies (Brown, 2012; Lee, 2013). The belief of using direct and indirect feedback strategies concurrently is also related to error types, which are viewed as a mediating role in teachers' choice of feedback strategies (Ferris, 2002; Bitchener and Ferris, 2012). As Han mentioned, there were various types of errors in students' writing and teachers' feedback strategies should be responsive. Teachers' beliefs in an integration of direct and indirect feedback demonstrate that teachers deciding the use of feedback is not a spontaneous practice, but is mediated by their students and error types (Lee, 2014).

### Beliefs About Feedback Orientation

Whereas there was no agreement in NES teachers' beliefs regarding feedback orientation, their NNES peers mostly believed in using negative feedback comments. Among the NES teachers, Bruce and Jason favored negative comments, and a combination of positive and negative feedback, respectively. The other two teachers, George and Christine, supported the use of positive feedback. Teachers' beliefs in positive feedback are important, for they probably recognized the merits of positive comments in enhancing students' motivation in writing. As Ellis (2009) argued, teachers should provide students with positive comments, as they give them affective support and boost their motivation. In contrast to the NES teachers, three NNES counterparts supported the use of negative feedback, stating that it could alert students to their problems and weaknesses in writing. Several studies have claimed that it enables students to have a deep insight into their inadequacies in learning (Kumar and Stracke, 2007).

The finding that NNES teacher placed emphasis on negative feedback is not surprising, as in Chinese traditional culture, negative feedback is considered to be “忠言” (the earnest advice) (Xu, 2017). As an old Chinese proverb goes, “良药苦口利于病, 忠言逆耳利于行” (bitter medicine cures illness and unpalatable advice benefits conduct). Negative feedback is deemed to demonstrate care and love from teachers for students, with teachers' provision of negative feedback implying that teachers are strict with their students. Negative feedback is greatly advocated and encouraged in Chinese cultural values and can be demonstrated by many Chinese old saying including “教不严, 师之惰” (if a teacher is not strict in teaching, it is his/her laziness) and “严师出高徒” (strict teachers produce excellent students). Thus, it is not a tradition for teachers to praise their students in Chinese education (Wang, 2015).

## Conclusion

Anchored in Chinese University EFL writing settings, this study investigated written feedback from the perspective of teachers' beliefs. It shows that NES and NNES teachers held a set of written feedback beliefs in different dimensions, and there were similarities and differences between the two groups of teachers' beliefs. For example, NES and NNES teachers reached a general agreement in feedback focus: Almost all of them emphasized that more attention should be paid to global issues. However, a marked difference was observed in their beliefs regarding feedback scope. Specifically, three NES teachers supported focused feedback, whereas three NNES teachers said that they believed in comprehensive feedback.

As one of the first attempts to investigate how teachers with different L1 view written feedback in a specific context, our study advanced the current body of literature on L2 teachers' beliefs of written feedback. As mentioned earlier, few studies of this topic took teachers' sociocultural/language backgrounds into consideration. As a result, setting teachers' L1 (English vs. Chinese) as a variable, this study fills an important niche and provides a nuanced understanding of L2 teachers' written feedback beliefs by comparing such two groups of teachers' conceptualizations.

In addition, this study also affords several important pedagogical implications. Firstly, the results of this study are probably beneficial for teacher educators and L2 writing researchers. To be specific, our results would offer them useful information in terms of designing effective and targeted training programs about how to provide written feedback in the Chinese EFL context and other similar contexts, which may facilitate L2 teachers' feedback provision and improve the effectiveness of such a teaching practice. As Borg (2015) argued, examining teacher belief is a prerequisite for useful and valuable teacher training programs. Furthermore, as some researchers have recommended, L2 teachers should provide focused written feedback, focus on both local and global issues, as well as balance positive and negative comments (Ferris, 2014; Lee, 2017). Unfortunately, such recommendations were not fully manifested in our NES and NNES teachers' beliefs. In this sense, teacher education programs regarding feedback provision should be held and offered by Chinese universities. It is necessary that teachers proactively participate in these programs in different forms such as workshops and seminars. By doing so, they can develop their pedagogical knowledge regarding feedback provision, and have a better understanding of this practice. In addition to the training programs, NES and NNES teachers can become self-trainers. Specifically, they can access to the research papers, discuss feedback provision with teacher educators and L2 writing researchers, and attend international/domestic academic conferences regarding L2 writing and writing assessment. Such practices would reshape and update their beliefs about how to provide feedback and improve their feedback literacy. Finally, NES and NNES participants in this study complained the contextual constraints including large-size classes and tight teaching schedules in the interviews. This means that L2 teachers did not have full autonomy while implementing their teaching practices. To empower them, administrators in Chinese universities might need to adopt measures to reduce teachers' workloads and large-class sizes to provide teachers with more time to reflect on their own feedback practices and acquire new knowledge about how to provide effective feedback.

Understandably, this study has some limitations. Firstly, it recruited only four NES and four NNES EFL teachers, respectively. The small sample of population would restrict the generalizations of the findings of this study. Thus, further studies should enlarge the sampling size to increase the generalizability of research findings. Moreover, given the research purposes, this study mainly employed semi-structured interviews to elicit NES and NNES teachers' written feedback conceptions. In this sense, further studies need to utilize other methods to collect data such as students' writing samples, stimulated recall interviews, reflection journals, and think-aloud. The data from these methods can triangulate with our findings and enable us to establish a comprehensive picture about how the two groups of teachers theorize written feedback in their particular teaching settings. Lastly, this study compared the two groups of teachers' beliefs within a relatively short timeframe. Considering that teacher belief is not a static construct but a dynamic one (Borg, 2003, 2015), longitudinal studies are warranted to trace any changes in their written feedback beliefs given that writing is a complex endeavor (Bitchener and Storch, 2016; Hyland and Hyland, 2019; Qin and Zhang, 2019; Zhang and Zhang, 2021).

## Data Availability Statement

The original contributions presented in the study are included in the article/supplementary material, further inquiries can be directed to the corresponding author/s.

## Ethics Statement

The studies involving human participants were reviewed and approved by University of Auckland Ethics Committee on Human Participants. The patients/participants provided their written informed consent to participate in this study.

## Author Contributions

XC conceived of the initial idea, designed the study, collected and analyzed the data, and drafted the manuscript. LZ fine-tuned, revised, proofread, and finalized the manuscript for submission. All authors contributed to the article and approved the submitted version.

## Funding

This study was supported by PhD startup research funding from Hubei University of Technology (No. 00491).

## Conflict of Interest

The authors declare that the research was conducted in the absence of any commercial or financial relationships that could be construed as a potential conflict of interest.

## Publisher's Note

All claims expressed in this article are solely those of the authors and do not necessarily represent those of their affiliated organizations, or those of the publisher, the editors and the reviewers. Any product that may be evaluated in this article, or claim that may be made by its manufacturer, is not guaranteed or endorsed by the publisher.

## Footnotes

^1^TEM-4 is a large-scale and well-established test to evaluate English major students' English proficiency in mainland China.

## Appendix A

### Interview Guide

#### Section 1. Teachers' Personal Background Information

1. Could you please tell me what degree you hold? And in which major?
2. Could you please tell me your experience of learning English writing? writing? In particular, how your teachers give feedback on your writings?
3. Please tell me your experience of teaching English, especially teaching Englishwriting?
4. Is your teaching of English writing and giving feedback similar or different fromyour teachers'?
5. Have you ever received any trainings on how to teach English writing and givefeedback?

#### Section 2. Teachers' Specific Beliefs About Written Feedback

6. In your opinion, is it important for teachers to provide feedback on students' writings? Why?
7. Do you think teachers should provide feedback comprehensively or selectively? Why?
8. In your opinion, what areas teachers should focus on in their written feedback? Why?
9. Do you think how teachers should indicate errors in students' writings? Why?
10. Do you think teachers should present feedback directly or indirectly? Why?
11. Do you think teachers should provide their feedback in a positive or negative way? Why?
12. Could you please tell me your ideal way to provide feedback on students' writings?
13. Do you have any comments/recommendations/problems concerning written feedback provision?
